# Spontaneous Intraperitoneal Rupture of Bladder Diverticulum: A Rare Cause of Peritonitis

**DOI:** 10.1155/2020/8880748

**Published:** 2020-12-01

**Authors:** Ahmed Ibrahimi, Adil Kallat, Idriss Ziani, Hachem El Sayegh, Lounis Benslimane, Yassine Nouini

**Affiliations:** ^1^Department of Urology A, Ibn Sina University Hospital, Faculty of Medicine and Pharmacy, Mohammed V University in Rabat, Rabat, Morocco; ^2^Provincial Hospital Center of Azilal, Azilal, Morocco

## Abstract

Spontaneous rupture of acquired bladder diverticulum in an adult patient is an extremely rare pathological entity with only 15 cases reported in the available literature. Etiologies are dominated by bladder outlet obstruction, represented mainly by benign prostatic hypertrophy (BPH) in elderly men. Clinical presentation is nonspecific, leading to delayed diagnosis and treatment. Surgical management is the standard approach for intraperitoneal rupture of bladder diverticulum. The prognosis depends on the earliness of treatment, associated comorbidity, and the nature of underlying diseases. Herein, we report a rare case of a 65-year-old male patient, who presented a spontaneous rupture of a large acquired bladder diverticulum, secondary to acute urinary retention complicating benign prostatic hypertrophy. The diagnosis was suspected in the presence of generalized peritonitis associated with lower urinary tract symptoms (LUTS) and was confirmed accurately and promptly by a computed tomography (CT) of the abdomen and pelvis. The patient underwent a successful surgical excision of the diverticulum and bladder repair without delay. The postoperative course was uneventful, and he was discharged from the hospital without any complication.

## 1. Introduction

Spontaneous rupture of acquired bladder diverticulum in an adult patient is a very rare pathological entity; to the best of our best knowledge, only 15 cases have been reported in the literature, and we add case 16 [1–3]. A bladder diverticulum is a mucosal protrusion through the detrusor muscle defect, composed of urothelial lining without a muscular layer [2]. It is most often secondary to bladder outlet obstruction such as benign prostate hyperplasia, repetitive infections, and iatrogenic causes [2]. The nonspecificity of its symptoms can delay diagnosis and treatment and can lead to serious complications or even death [4]. Surgical management remains the standard approach for intraperitoneal bladder rupture [2, 3, 5, 6]. However, conservative management can be successfully performed in selected patients with favourable characteristics [1, 7].

Here, we report a rare case of spontaneous intraperitoneal rupture of bladder diverticulum secondary to an episode of acute urine retention complicating benign prostatic hypertrophy, described in a 65-year-old male patient with a history of untreated BPH, and which was successfully managed by a prompt diagnosis and surgical treatment. Through this report, we aimed to highlight this rare cause of acute peritonitis, as well as to discuss the different clinical, etiological, and therapeutic aspects of this surgical emergency.

## 2. Case Presentation

A 65-year-old man, with a history of benign prostatic hypertrophy (BPH) not followed for 2 years, without other medical complaints, was admitted to the emergency for acute urinary retention (AUR); he underwent urethral catheterization and returned home with alpha-blocker therapy, and two days later, he represented to the emergency department with diffuse acute abdominal pain, especially in the hypogastric region, nausea and vomiting, associated with generalized weakness and fever.

Physical examination of the abdomen revealed diffuse tenderness associated with muscle guarding and rebound pain especially in the hypogastric area, without distended urinary bladder; the digital rectal examination (DRE) was painful and found a firm and enlarged prostate gland. His temperature was 38°C, with blood pressure of 125/62 mmHg, heart rate of 94 beats/min, and respiratory rate of 17 breaths/min.

Laboratory testing revealed hemoglobin of 12.8 g/dL, platelet count of 520,000/mm^3^, increase in serum urea of 0.73 g/dL and in serum creatinine of 27 mg/dL, with elevated of white blood cell (WBC) of 17,300/*μ*L, and C-reactive protein (CRP) of 96.2 mg/dL. After conditioning, an intravenous antibiotic was administered, and a urinary catheterization had given 80 mL of cloudy urine, and the urine test strip was positive.

**Figure 1:**
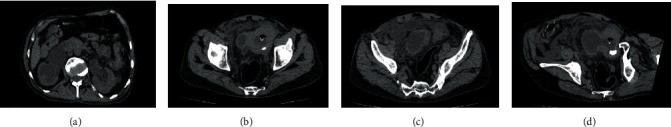
Axial view of CT scan showing (a) bilateral hydroureteronephrosis. (b, d) Empty bladder, associated to a large full bladder diverticulum, with bladder stone and bilateral ureteral dilation. (c) Small amount of free intraperitoneal fluid leaking from a defect in the bladder diverticulum wall.

**Figure 2:**
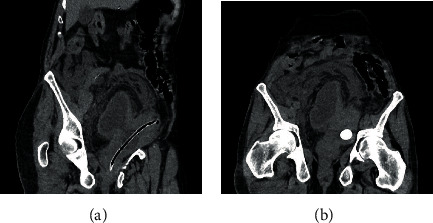
(a, b) Coronal oblique reconstruction of the CT scan showing collapsed bladder with a large diverticulum arising from the right superolateral bladder wall, with a large bladder stone and Foley catheter in place, associated to a small amount of free fluid in the peritoneal cavity and around the diverticulum, with an enlarged prostate gland.

**Figure 3:**
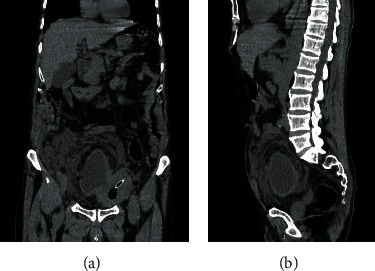
(a) Coronal reconstruction of the CT scan showing empty bladder with small amount of intra-abdominal fluid leaking from a ruptured large bladder diverticulum and perivesical fat infiltration. (b) Sagittal reconstruction of the CT scan showing a large full ruptured bladder diverticulum with fluid collection around it and ureteral dilation.

Ultrasonography (US) showed bilateral hydroureteronephrosis with empty bladder containing a small amount of urine and a large bladder stone; these findings are associated with the presence of a large full bladder diverticulum and a small amount of free peritoneal fluid. CT of the abdomen and pelvis without contrast (due to the kidney failure) confirmed the presence of a small amount of free fluid in the abdominal cavity, associated with massive fluid accumulation in the right anterolateral paravesical space, between the bladder and the anterior abdominal wall (Figures 1(b)–1(d), 2, and 3), and a ruptured large bladder diverticulum arising from the right superolateral wall of the urinary bladder (Figures 1(c) and 3) with bilateral hydroureteronephrosis (Figure 1(a)), large bladder stone (Figures 1(b), 1(d), and 2(b)) and enlarged prostate gland measuring 63 g (Figure 2). Clinical presentation and imaging findings lead to the diagnosis of acute peritonitis secondary to spontaneous intraperitoneal perforation of bladder diverticulum.

The patient was transferred to the operation theater, and an emergency midline laparotomy was performed, revealing an intraperitoneal rupture of a large bladder diverticulum which was located on the right superolateral wall of the bladder, with fluid collection around it, and free purulent fluid in the abdominal cavity; there was no evidence of pathologic lesions at the site of the injured diverticulum and peritoneum. A copious saline lavage of the peritoneal cavity was done, the diverticulum was excised, the bladder stone was removed, and the bladder defect was repaired in two layers with continuous 2/0 Vicryl sutures. Successful repair of the bladder defect was confirmed intraoperatively by the absence of leakage when filling the bladder, and a pelvic drain left in situ at the end of intervention.

The postoperative course was uneventful. The inflammatory markers and renal function showed decreases over the following days to reach its normal level. Cystogram performed at the eighth day showed a watertight bladder.

The patient was discharged from the hospital at the 10th day with an indwelling bladder catheter, and a transurethral prostatic resection (TUPR) was planned after 6 weeks.

## 3. Discussion

Urinary bladder ruptures are in most cases secondary to blunt abdominal trauma, pelvic fractures, or iatrogenic causes, and 96.6% of all cases of bladder ruptures are posttraumatic [8, 9]. A spontaneous, nontraumatic urinary bladder rupture is a very rare surgical emergency, with an incidence of 1 : 126,000 and a mortality rate of 47% [5].

Bladder rupture can be classified into intraperitoneal or extraperitoneal types depending on the defect location, which occurs usually in the bladder dome which is the weakest point of the bladder wall, causing an intraperitoneal bladder rupture [5, 10, 11]. Common predisposing factors for spontaneous bladder rupture are associated with either increased bladder pressure or decreased strength of the bladder wall or its combination [12]. In the available literature, only a few cases of spontaneous bladder rupture were reported; the common causes are malignancy, pervious pelvic radiotherapy, neuropathic bladder dysfunction, bladder inflammation or infection, bladder outflow obstruction [4, 5, 10, 12], alcohol intoxication (binge drinking) [13], female pelvic organ prolapse, and postpartum [10]; it can be also associated with extravesical pathology such as appendiceal abscesses, tuboovarian abscesses, divertitular disease, or even surgical drain placed alongside the bladder [14], or in the rare cases secondary to ruptured congenital or acquired bladder diverticulum [1–3].

The clinical presentation is nonspecific, including painful abdominal distension, decreased urine output, and voiding complaints [2, 11], and in some cases, it can take on the appearance of acute peritonitis with sepsis, oliguria, and acute renal failure due to reabsorption of creatinine in the urinary ascites [5, 9]; this atypical presentation can delay diagnosis and treatment and lead to serious, life-threatening complications, especially in the case of spontaneous intraperitoneal rupture of bladder diverticulum [4]. In the present case, both bladder diverticulum and bladder stone associated to renal failure are the complications of BPH, and intraperitoneal bladder rupture seems to be the result of a combination of sudden increase in intravesical pressure due to AUR, and a decrease of the strength of the bladder wall due to bladder diverticulum.

Considering its rarity, the diagnosis should be suspected in the presence of the past urological history of risk factors suggestive of bladder rupture, associated with symptoms of acute abdomen. The positive diagnosis is based on CT cystography which remains the gold standard for the diagnosis, and it is most sensitive for detecting the presence of bladder perforation than ultrasound or conventional CT [15]. CT cystography compared to retrograde cystography is less invasive with comparable findings and has an additional advantage of exploration the whole abdominal cavity [15]. Ultrasound can also make the diagnosis by instillation of saline and air into the bladder [11]. Cystoscopy is not recommended but can be used to place the urethral catheter in the cases of urethral stricture or false passage [16]. In our case, retrograde cystography was not performed because of the urinary tract infection, CT scan without contrast allowed us to make the diagnosis, and the exam positivity is due to the defect location which was not plugging during the radiological examination, and the large size of the diverticulum which was always filled with urine despite its perforation, probably because the diverticulum wall was not completely ruptured and because of the small size of the defect.

When diagnosed, treatment depends on type of bladder rupture as well as the presence of an underlying pathology which may require additional treatment [6]. Intraperitoneal rupture of a bladder diverticulum requires immediate surgical repair to avoid serious complications, it can be made by laparotomy or laparoscopically in selected patients [3], and it consists of exploring the whole abdominal cavity, excising the diverticulum, repairing the bladder defect, and draining the abdominal fluid [4, 8, 13, 14]. Conservative management without surgery can be successfully used in carefully selected patients with only urinary catheterization and antibiotics, with or without percutaneous peritoneal drainage [1, 4, 7].

After the acute episode, a special consideration must be given to the treatment of the associated diseases to prevent recidive. In our case, conservative management has not been attempted because of the presence of clinical and biological sepsis, and the presence of bladder stone requiring surgical treatment. The uneventful postoperative course without complication can be attributed to the prompt diagnosis and treatment and the absence of other associated pathologies.

## 4. Conclusion

Spontaneous intraperitoneal rupture of bladder diverticulum is a rare surgical emergency, and diagnosis should be suspected without delay in the presence of acute abdomen associated with lower urinary tract symptoms. Prevention requires better management of underlying pathologies and risk factors, and prompt and accurate diagnosis is the key for successful management.
